# The Potential Related Genes and Mechanisms Involved in Improving the Treadmill Exercise Ability of APP/PS1 Mice

**DOI:** 10.3390/ijms251910244

**Published:** 2024-09-24

**Authors:** Zhe Zhao, Xingqing Wu, Weijia Wu, Yingzhe Tang, Xiangyuan Meng, Mei Peng, Changfa Tang, Lan Zheng, Wenfeng Liu

**Affiliations:** Hunan Provincial Key Laboratory of Physical Fitness and Sports Rehabilitation, Hunan Normal University, Changsha 410012, China; zhaozhe@hunnu.edu.cn (Z.Z.); wuxingqing2004@163.com (X.W.); 13080535713@163.com (W.W.); tangyingzhe92@163.com (Y.T.); mengxiangyuan188@163.com (X.M.); pengmei@hunnu.edu.cn (M.P.); lanzheng@hunnu.edu.cn (L.Z.)

**Keywords:** Alzheimer’s disease, exercise, skeletal muscle, exercise ability

## Abstract

Alzheimer’s disease (AD) causes a decline in skeletal muscle function, which can further exacerbate the cognitive dysfunction of patients with AD. It has been widely established that exercise improves AD brain pathology, but the role of skeletal muscle in AD is still poorly understood. In this study, we investigated the effects of treadmill exercise on the exercise ability of APP/PS1 transgenic AD mice and explored potential gene expression changes in their skeletal muscle. The APP/PS1 mice were subjected to a treadmill exercise for 12 weeks, followed by the Morris water maze and the open field test. After behavioral experiments, the changes in morphology, area, collagen fiber deposition, and ultrastructure of the skeletal muscle were determined; the balance of skeletal muscle protein synthesis and decomposition was analyzed; and changes in gene expression were investigated using RNA-Seq. We found that this exercise strategy can promote the learning and memory abilities of AD mice, reduce their anxiety-like behavior, improve their exercise ability, alleviate skeletal muscle atrophy, and optimize the microstructure. It can also enhance skeletal muscle protein synthesis and decomposition and improve several signaling pathways, such as the JAK–STAT, Wnt, and NOD-like receptors while decreasing calcium, cAMP, cGMP–PKG, and other signaling pathways. Six KEGG enrichment signaling pathways were downregulated and five signaling pathways were upregulated in the AD mice compared with wild-type mice, and these pathways were precisely reversed after the treadmill exercise. The expression of transcription factors such as Fosb and Egr1 in the skeletal muscle of AD mice decreased, followed by a decrease in the regulated target genes Socs1, Srrm4, and Il1b, a trend that was reversed following the exercise intervention. After exercise, AD mice exhibited a similar gene expression to that of wild-type mice, indicating enhanced exercise ability. The potential regulatory pathways and related genes identified in this study provide valuable insights for the clinical management and treatment of AD.

## 1. Introduction

Alzheimer’s disease (AD) is a common neurodegenerative disease with symptoms such as memory decline, cognitive dysfunction, personality change, and language disorders [1]. To date, AD has affected millions of people worldwide, with its prevalence estimated to reach 115 million by 2050 [2]. AD severely affects brain health and reduces the quality of life of patients [3,4], placing a huge burden on their families and society. Currently, it is a major health risk for the aging population.

AD presents classic pathological features, mainly in the brain, such as the abnormal aggregation of amyloid β-protein (Aβ) plaques; phosphorylated tau (p-tau) caused by neurofibrillary tangles; and microglia overactivation, neuron loss, and synaptic dysfunction [5,6,7,8]. However, current drug treatments for the pathological features of the brain have failed one after another. In a previous study involving older Mexican adults, Salinas-Rodriguez et al. revealed a significant longitudinal association between sarcopenia, mild cognitive impairment, and cognitive function [9]. Hu et al. found that more severe cognitive impairment in older Chinese adults was associated with sarcopenia [10]. Animal experiments have also revealed that neuromuscular dysfunction occurs before cognitive impairment in the AD model mice [11,12]. Progressive and accelerated neurogenic sarcopenia precedes the presentation of AD symptoms and may, thus, be used to predict cognitive deficits in AD [13]. Some skeletal muscle mechanisms may contribute to central metabolic function [14]. The above findings suggest a peripheral organ pathogenesis of AD and that a decline in skeletal muscle function is a primary cause of AD cognitive dysfunction; therefore, intervention in the skeletal muscle function may be an effective method to treat AD.

In their review, Liu et al. selected 17 preclinical reports after screening 7638 studies. Of those, four studies reported that muscle atrophy and injury impair memory and neurons in the brains of rodents with or without AD, whereas six studies revealed that myostatin knockdown can relieve symptoms of AD [15]. This further supports muscle-specific therapy as a potential clinical strategy to prevent cognitive dysfunction. However, currently, experimental studies on AD skeletal muscle are relatively limited. Exercise is one of the most effective and healthy ways to promote skeletal muscle function. Although the ameliorative effects of exercise on AD brain pathology have been widely confirmed [16,17], few studies exist on the effects of exercise on AD skeletal muscle. In this study, we investigated the presence of skeletal muscle atrophy in APP/PS1 transgenic AD mice, observed the effects of treadmill exercise on the exercise ability and skeletal muscle function of AD mice and then using RNA-Seq explored comprehensively the changes in skeletal muscle gene expression. The results provide a strong basis for the new direction of skeletal muscle–brain interaction in AD.

## 2. Results

### 2.1. Treadmill Exercise Improved Memory and Reduced the Anxiety Behavior of AD Mice

**Figure 1:**
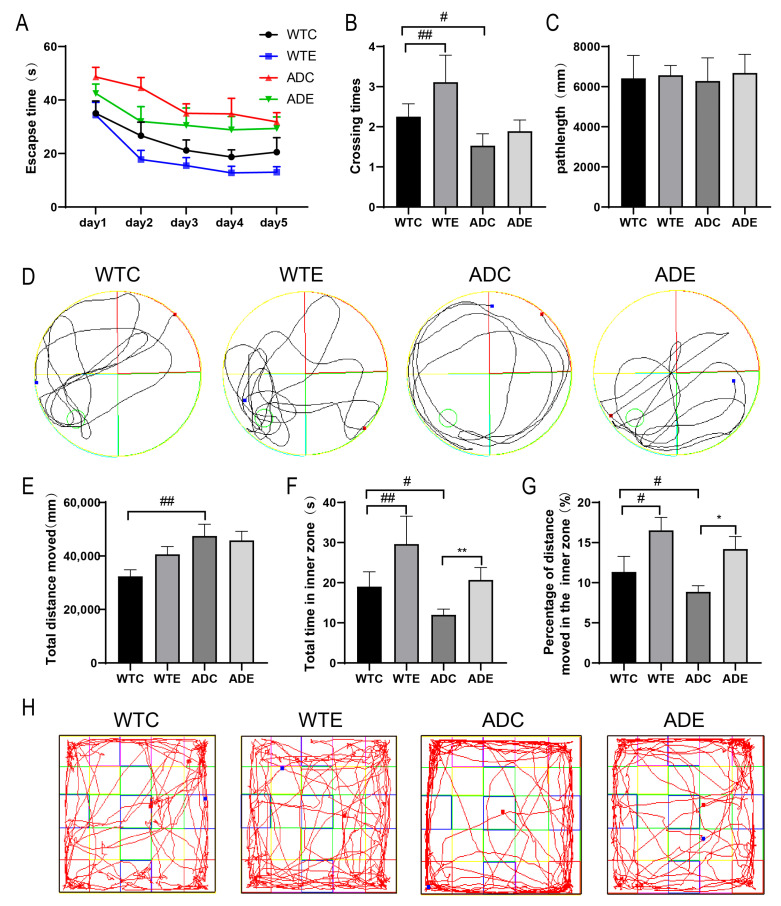
Treadmill exercise improved memory and reduced the anxiety behavior of AD mice: (**A**) changes in the latency period observed using the Morris water maze; (**B**) numbers of times the platform was crossed in the Morris water maze; (**C**) total swimming distance in the Morris water maze; (**D**) swimming tracks in the Morris water maze, with the circle in the southwest quadrant showing the location of the platform; (**E**) total distance traversed in the open field test; (**F**) time taken to traverse the central area in the open field test; (**G**) percentage of distance in the central area in the open field test; (**H**) representative images of moving tracks in the open field test, in which the small squares were added when statistical analysis was performed (*n* = 10). Data are expressed as the means ± SD; # *p* < 0.05 and ## *p* < 0.01 compared to the WTC group; * *p* < 0.05 and ** *p* < 0.01 compared to the ADC group.

As shown in Figure 1A, with an increase in learning time, the latency period decreased among the four groups of mice, while AD mice exhibited the longest latency period. However, the exercise intervention resulted in a shorter latency period in both AD mice and wild-type mice. After removing the platform, the number of times WTE mice traversed the platform significantly increased, while that of the ADC group significantly decreased compared with the WTC group mice; the number of times AD mice traversed the platform was improved to a certain extent after the exercise intervention (Figure 1B). After removing the platform, no significant difference was observed in the total swimming distance in the water maze among each group of mice (Figure 1C), suggesting that the abovementioned differences did not contribute to differences in their swimming ability. As shown in Figure 1E, the total moving distance of AD mice in the open field increased significantly, while their time and distance in the central area decreased significantly (Figure 1F,G). Additionally, wild-type mice were more dispersed in the open field, while AD mice were more concentrated at the edges and corners; however, the exercise intervention altered this trend (Figure 1H). Since anxious mice are more inclined to move around the edges of the open field [18], this suggests that exercise reduces anxiety in mice. These data suggest that AD mice exhibited cognitive dysfunction and anxiety behavior, which were alleviated after the treadmill exercise.

### 2.2. Treadmill Exercise Improved the Exercise Ability and Alleviated the Skeletal Muscle Atrophy in AD Mice

**Figure 2:**
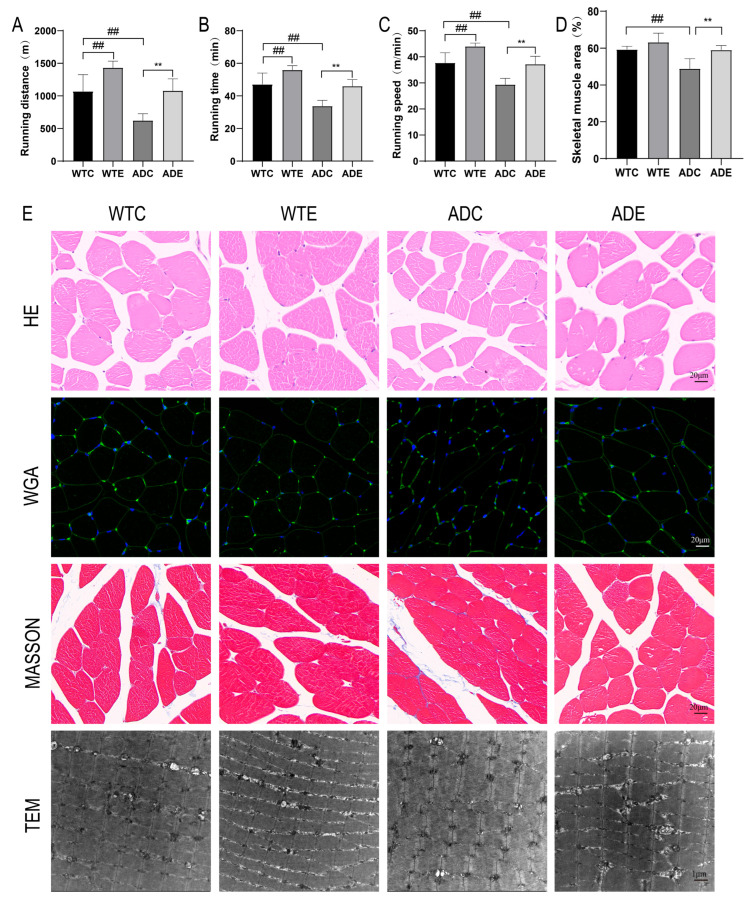
Treadmill exercise improved the exercise ability and alleviated the skeletal muscle atrophy of AD mice: (**A**) total running distance until exhaustion; (**B**) total running time until exhaustion; (**C**) speed at the point of exhaustion (*n* = 4); (**D**) percentage of HE-stained skeletal muscle cross-sectional area; (**E**) HE staining, WGA staining, Masson staining, and representative microscopic images under a transmission electron microscope for observed skeletal muscle (*n* = 3). Data are expressed as the means ± SD; ## *p* < 0.01 compared to the WTC group; ** *p* < 0.01 compared to the ADC group.

AD is a neurodegenerative disease. Thus, after the behavioral assessment of the brain, we tested the exercise ability of mice to explore changes in skeletal muscle function. It was found that running distance, running time, and speed at exhaustion were significantly shorter in the AD mice than in wild-type mice, and treadmill exercise significantly improved the running distance, running time, and speed at the point of exhaustion of both wild-type and AD mice (Figure 2A–C). This indicates that treadmill exercise improved the exercise ability of mice. The changes in the skeletal muscle morphology were further analyzed using hematoxylin–eosin (HE) staining, which revealed that the skeletal muscle fibers were more rounded in wild-type mice. By contrast, atrophy was observed in the muscle fibers of AD mice, which decreased in area and exhibited sharp edges, whereas the area became larger and more rounded after exercise (Figure 2D,E). The wheat germ agglutinin (WGA) staining of the muscle cell membrane further confirmed this change. Masson staining revealed collagen fiber deposition in the skeletal muscle of AD mice, which was attenuated after treadmill exercise. Based on transmission electron microscopy images, the skeletal muscle fibers of wild-type mice exhibited regular arrangement, with the Z lines connected in a straight line, whereas the skeletal muscle fibers of AD mice exhibited chaotic arrangement, with intersecting and twisted Z lines that did not converge in a neat straight line. However, after the intervention, regular arrangement and the convergence of the Z lines were observed in the skeletal muscle fibers. These data point to skeletal muscle atrophy, collagen fiber deposition, and disorganized skeletal muscle fibers in the AD mice, resulting in decreased exercise ability, while the treadmill exercise improved the exercise ability of AD mice and reduced their skeletal muscle atrophy.

### 2.3. Treadmill Exercise Increased the Expression of Genes and Proteins Related to Skeletal Muscle Protein Synthesis in the AD Mice

**Figure 3:**
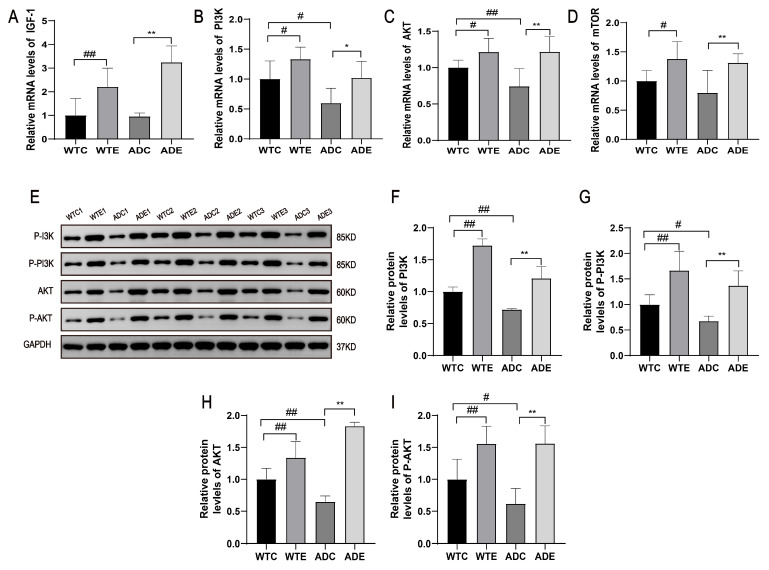
Treadmill exercise increased the expressions of genes and proteins involved in skeletal muscle protein synthesis in the AD mice: (**A**) mouse skeletal muscle IGF-1 mRNA expression; (**B**) mouse skeletal muscle PI3K mRNA expression; (**C**) mouse skeletal muscle AKT mRNA expression; (**D**) mouse skeletal muscle mTOR mRNA expression; (**E**) representative images of Western blotting of PI3K, p-PI3K, AKT, and p-AKT; (**F**) relative expression of PI3K protein; (**G**) relative expression of p-PI3K protein; (**H**) relative expression of AKT protein; (**I**) relative expression of p-AKT protein (*n* = 6). Data are expressed as the means ± SD; # *p* < 0.05 and ## *p* < 0.01 compared to the WTC group; * *p* < 0.05 and ** *p* < 0.01 compared to the ADC group.

Skeletal muscle atrophy is caused by an imbalance between protein synthesis and degradation; thus, we investigated protein synthesis-related genes and observed the downregulation of IGF-1, PI3K, AKT, and mTOR gene expressions in the skeletal muscle of AD mice, whereas exercise elevated the expressions of these genes (Figure 3A–D). Through further analysis, we found that PI3K, p-PI3K, AKT, and p-AKT protein expression levels were reduced in the AD mice and elevated after exercise. These data suggest that skeletal muscle protein synthesis is reduced in the AD mice, while the treadmill exercise promotes skeletal muscle protein synthesis in the AD mice.

### 2.4. Treadmill Exercise Increased the Expressions of Genes and Proteins Related to Skeletal Muscle Protein Degradation in the AD Mice

**Figure 4:**
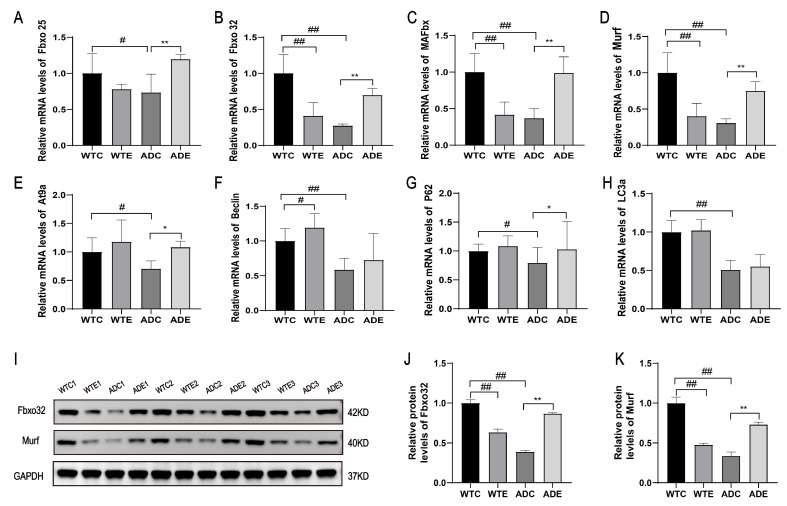
Treadmill exercise increased the expressions of genes and proteins related to skeletal muscle protein degradation in the AD mice: (**A**) mouse skeletal muscle Fbxo 25 mRNA expression; (**B**) mouse skeletal muscle Fbxo 32 mRNA expression; (**C**) mouse skeletal muscle MAFbx mRNA expression; (**D**) mouse skeletal muscle Murf mRNA expression; (**E**) mouse skeletal muscle At9a mRNA expression; (**F**) mouse skeletal muscle Beclin mRNA expression; (**G**) mouse skeletal muscle p62 mRNA expression; (**H**) mouse skeletal muscle LC3 mRNA expression; (**I**) representative images of the Western blotting of Fbxo 32 and Murf; (**J**) relative expression of Fbxo 32 protein; (**K**) relative expression of Murf protein (*n* = 6). Data are expressed as the mean ± SD; # *p* < 0.05 and ## *p* < 0.01 compared to the WTC group; * *p* < 0.05 and ** *p* < 0.01 compared to the ADC group.

The ubiquitin–proteasome system (UPS) and the autophagy lysosomal pathway (ALP) contribute to more than 80% of protein degradation [19], and we found that UPS markers such as Murf and Fbxo 32 were significantly downregulated in the skeletal muscle of AD mice (Figure 4A–D,I–K), as well as ALP-related factors At9a and LC3a (Figure 4E–H), while the treadmill exercise reversed this trend. Murf and Fbxo 32 were also significantly downregulated in the WTE group mice (Figure 4A–D,I–K). The above results indicate that the WTE group mice exhibited an increase in skeletal muscle protein synthesis and a decrease in degradation, while in the AD mice, both skeletal muscle protein synthesis and degradation decreased. However, after the intervention, an increase was observed in both skeletal muscle protein synthesis and degradation, suggesting that exercise may play a role in regulating new homeostasis of protein synthesis and degradation in the skeletal muscle of AD mice, leading to enhanced skeletal muscle health and, thus, improvement in exercise ability.

### 2.5. Treadmill Exercise Altered Gene Expression in the Skeletal Muscle of AD Mice

**Figure 5:**
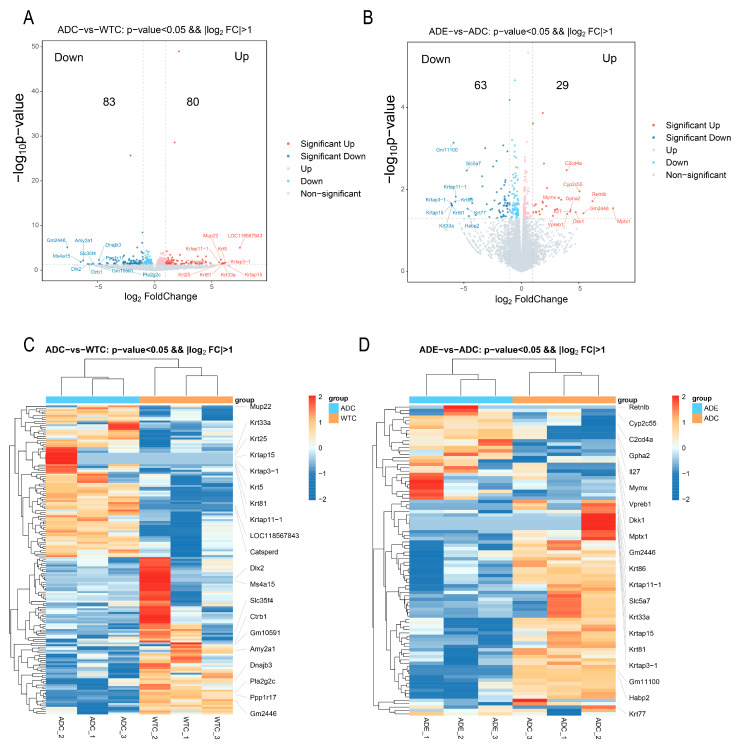
Treadmill exercise altered the gene expression in the skeletal muscle of the AD mice: (**A**) volcano plot of the DEGs in the ADC group versus the WTC group; (**B**) volcano plot of the DEGs in the ADE group versus the ADC group; (**C**) heatmap of the DEGs in the ADC group versus the WTC group; (**D**) heatmap of the DEGs in the ADE group versus the ADC group (*n* = 3).

For further analysis, we performed RNA-Seq to explore the characteristics of the gene changes. As shown in Figure 5A, 83 genes were significantly downregulated, and 80 genes were upregulated in the AD mice, compared to the WT mice. As shown in Figure 5B, exercise significantly downregulated 63 genes and upregulated 29 genes in the AD mice compared to those without exercise. Furthermore, a heatmap was used to demonstrate the DEGs, and it was found that gene expression was significantly different before and after the exercise intervention in the AD mice compared to the wild-type mice.

### 2.6. GO Enrichment Analysis of the DEGs

**Figure 6:**
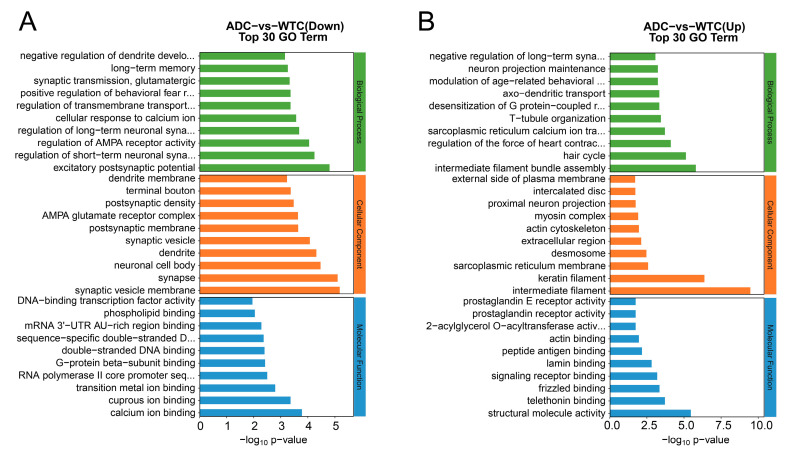

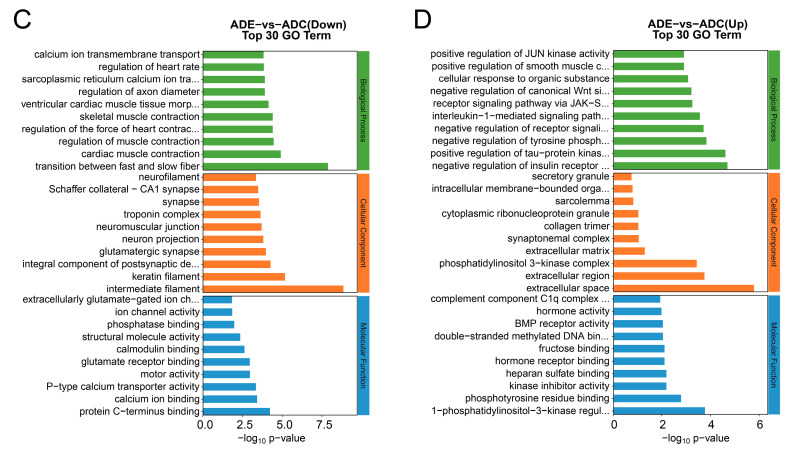
GO enrichment analysis of the DEGs: (**A**) GO enrichment analysis of downregulated genes in the ADC group compared to the WTC group; (**B**) GO enrichment analysis of upregulated genes in the ADC group compared to the WTC group; (**C**) GO enrichment analysis of downregulated genes in the ADE group compared to the ADC group; (**D**) GO enrichment analysis of upregulated genes in the ADE group compared to the ADC group (*n* = 3).

To further explore functions among the differential genes, we performed a GO enrichment analysis. The results of 30 enrichment bars indicate that compared to the WTC group, the enriched downregulated genes in the ADC group were mainly associated with biological processes such as the excitatory postsynaptic potential, regulation of short- and long-term neuronal synaptic plasticity, cellular response to calcium ion, regulation of transmembrane transporter activity, synaptic transmission, glutamatergic synaptic activity, and negative regulation of dendrite development; cell components such as the synaptic vesicle membrane, synapse, neuronal cell body, dendrite, synaptic vesicle, postsynaptic membrane, AMPA glutamate receptor complex, postsynaptic density, terminal bouton, and dendrite membrane; and molecular functions such as calcium ion binding, cuprous ion binding, G-protein beta-subunit binding, double-stranded DNA binding, sequence-specific double-stranded DNA binding, and DNA-binding transcription factor activity (Figure 6A). By contrast, the enriched upregulated genes were mainly involved in biological processes such as sarcoplasmic reticulum calcium ion transport, T-tubule organization, desensitization of the G protein-coupled receptor signaling pathway, axo-dendritic transport, neuron projection maintenance, and negative regulation of long-term synaptic potentiation; cell components such as the keratin filament, sarcoplasmic reticulum membrane, desmosome, extracellular region, actin cytoskeleton, proximal neuron projection, and external side of plasma membrane; and molecular functions such as signaling receptor binding, lamin binding, peptide antigen binding, actin binding, and 2-acylglycerol O-acyltransferase activity (Figure 6B). Meanwhile, compared to the ADC group, the enriched downregulated genes in the ADE group were mainly involved in biological processes such as the regulation of muscle contraction, skeletal muscle contraction, regulation of axon diameter, sarcoplasmic reticulum calcium ion transport, and calcium ion transmembrane transport; cell components such as the integral component of the postsynaptic density membrane, the glutamatergic synapse, neuron projection, neuromuscular junction, troponin complex, and synapse; and molecular functions such as protein C-terminus binding, calcium ion binding, glutamate receptor binding, calmodulin binding, structural molecule activity, and ion channel activity (Figure 6C). By contrast, the enriched upregulated genes were mainly attributed to biological processes such as positive regulation of tau-protein kinase activity, negative regulation of tyrosine phosphorylation of STAT protein, interleukin-1-mediated signaling pathway, JAK-STAT receptor signaling pathway, negative regulation of the canonical Wnt signaling pathway, and positive regulation of the JUN kinase activity; cell components such as the phosphatidylinositol 3-kinase complex, extracellular matrix, synaptonemal complex, collagen trimer, cytoplasmic ribonucleoprotein granule, the sarcolemma, and the intracellular membrane-bounded organelle; and molecular functions such as 1-phosphatidylinositol-3-kinase regulator activity, phosphotyrosine residue binding, kinase inhibitor activity, hormone receptor binding, double-stranded methylated DNA binding, BMP receptor activity, and complement-component C1q complex binding (Figure 6D).

### 2.7. KEGG Enrichment Analysis of the DEGs

**Figure 7:**
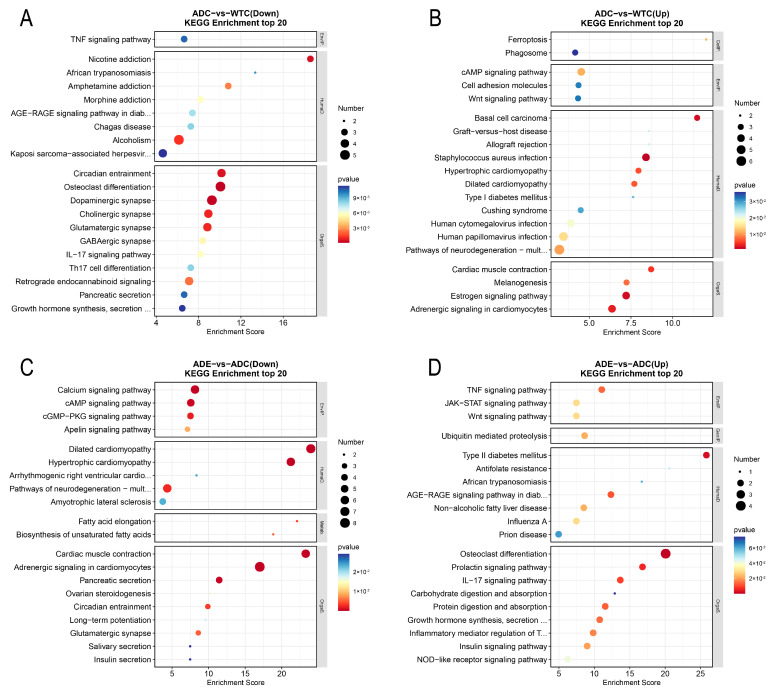
The KEGG enrichment analysis of the DEGs: (**A**) The KEGG enrichment analysis of downregulated genes in the ADC group compared to the WTC group; (**B**) the KEGG enrichment analysis of upregulated genes in the ADC group compared to the WTC group; (**C**) the KEGG enrichment analysis of downregulated genes in the ADE group compared to the ADC group; (**D**) the KEGG enrichment analysis of upregulated genes in the ADE group compared to the ADC group (*n* = 3).

To gain a deeper understanding of the function of DEGs, we also performed a KEGG enrichment analysis. The results of 20 enrichment bars indicate that compared to the WTC group, the enriched downregulated genes in the ADC group were mainly associated with the TNF signaling pathway; osteoclast differentiation; dopaminergic, cholinergic, and glutamatergic synapses; the IL-17 signaling pathway; and growth hormone synthesis and secretion (Figure 7A), which indicates deficiency in synaptic functions, immune functions, and cell growth in the AD mice skeletal muscle. By contrast, the enriched upregulated genes were associated with the pathways of neurodegeneration in multiple diseases, staphylococcus aureus infection, cAMP signaling pathway, cell adhesion molecules, and the Wnt signaling pathway (Figure 7B). Meanwhile, compared to the ADC group, the enriched downregulated genes in the ADE group were mainly involved in the cAMP signaling pathway, calcium signaling pathway, cGM–PKG signaling pathway, amyotrophic lateral sclerosis, and neurodegeneration pathways in multiple diseases (Figure 7C), while the enriched upregulated genes were associated with osteoclast differentiation, TNF, Wnt, NOD-like receptor, and IL-17 signaling pathways (Figure 7D). Notably, six signaling pathways involved in osteoclast differentiation (genes Fosb, Il1b, Socs1, and Socs3), IL-17 signaling (genes Fosb and Il1b), AGE-RAGE signaling in diabetic complications (genes Egr1 and Il1b), African trypanosomiasis (gene Il1b), and TNF signaling (gene Il1b and Socs3); and growth hormone synthesis, secretion, and action (genes Socs1 and Socs3) were downregulated in the AD mice compared to the WT mice, while they were upregulated after the treadmill exercise. Furthermore, five signaling pathways associated with dilated cardiomyopathy (genes Adcy8, Atp2a2, Myh7, Myl3, Ryr2, Tnnc1, and Tpm3); adrenergic signaling in cardiomyocytes (genes Adcy8, Atp2a2, Atp2b2, Myh7, Myl3, Ryr2, Tnnc1, and Tpm3); cardiac muscle contraction (genes Atp2a2, Myh7, Myl3, Ryr2, Tnnc1, and Tpm3); hypertrophic cardiomyopathy (genes Atp2a2, Myh7, Myl3, Ryr2, Tnnc1, and Tpm3); and cAMP signaling (genes Adcy8, Atp2a2, Atp2b2, Gria2, and Ryr2) were upregulated, while they were downregulated after the treadmill exercise.

### 2.8. Transcription Factor Analysis

**Figure 8:**
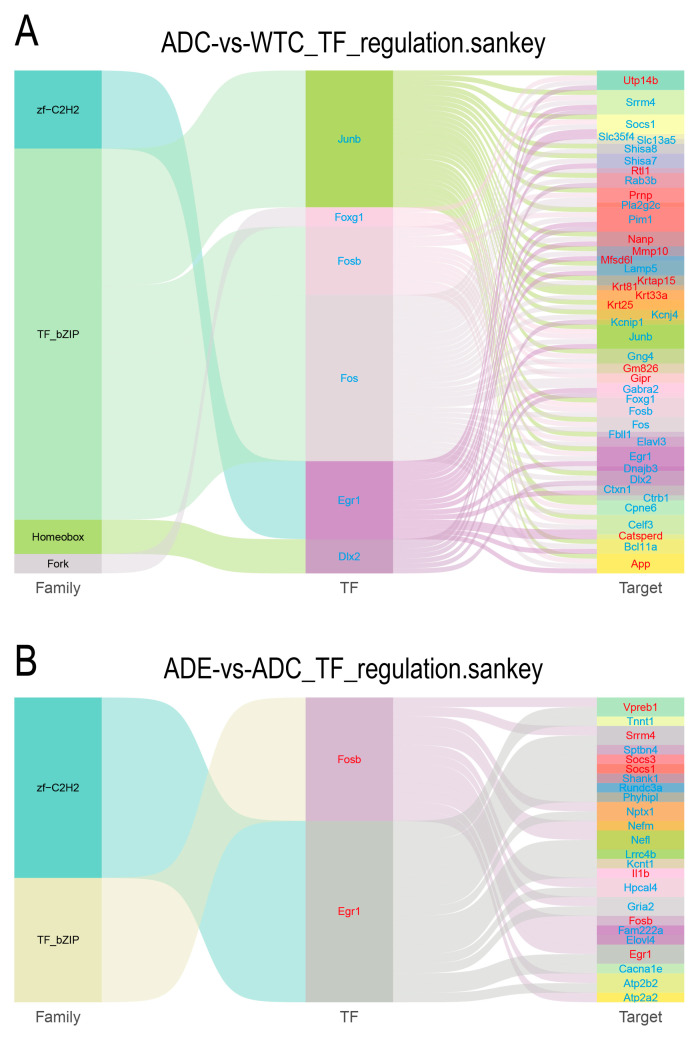
Differential transcription factor–target gene Sankey diagram. (**A**) Transcription factor changes in ADC group compared with WTC group. (**B**) Changes in transcription factors in ADE group compared with ADC group. From left to right: first column—transcription factor families; second column—differential transcription factors; third column—differential target genes. The middle line indicates the correspondence of transcription factor families, transcription factors, and target genes. The genes in blue are downregulated, while the genes in red are upregulated (*n* = 3).

Transcription factors (TFs) regulate intracellular gene expression and, thus, play a crucial role in all aspects of cellular physiology throughout the body [20]. Differential target genes corresponding to differential transcription factors were identified based on the list of associations between transcription factors and target genes, and a statistical map of the target genes of differential transcription factor families was drawn. Compared to the WT mice, six downregulated transcription factors were identified in the AD mice, corresponding to 42 differential target genes (Figure 8A). By contrast, after the intervention, two upregulated transcription factors were detected in the AD mice, corresponding to 24 differential target genes (Figure 8B). Notably, two transcription factors in the AD mice, Fosb and Egr1, were significantly downregulated compared to the WT mice, and their corresponding target genes, Srrm4, Socs1, Il1b, Fosb, and Egr1, were also significantly downregulated. Additionally, these transcription factors and target genes were upregulated after the treadmill exercise, thus highlighting their vital role in the decreased exercise ability of AD mice and their significant regulatory involvement in the enhanced exercise ability observed after the treadmill exercise.

## 3. Discussion

It has been widely established that an appropriate level of physical activity improves learning and memory abilities and reduces AD brain pathology. However, the effects of physical activity on AD skeletal muscle and exercise ability are rarely reported. We investigated the effects of treadmill activity on the exercise ability and skeletal muscle structure of APP/PS1 transgenic AD mice and used RNA-Seq to comprehensively explore the gene expression changes in skeletal muscle. The main findings are as follows: (1) A 12-week treadmill exercise regimen attenuated learning memory impairment and anxiety-like behavior in the AD mice. (2) The diminished exercise ability of AD mice was enhanced after the treadmill exercise. (3) Skeletal muscle atrophy, collagen fiber disposition, and disorganized muscle fibers were observed in the AD mice, whereas the treadmill exercise resulted in improvement in the skeletal muscle structure of AD mice. (4) Both skeletal muscle protein synthesis and degradation were reduced in the AD mice, whereas running increased the level of protein synthesis and breakdown in the AD mice. (5) Gene expression was significantly different in the skeletal muscle of AD mice compared to that of wild-type mice, pointing to the significant effect of the treadmill exercise on the gene expression of the skeletal muscle of AD mice. (6) Six KEGG-enriched signaling pathways were downregulated, and five were upregulated in the AD mice compared to the wild-type mice, whereas the expression levels of these pathways were reversed after the treadmill exercise. (7) Two transcription factors, Fosb and Egr1, as well as their corresponding target genes Srrm4, Socs1, Il1b, Fosb, and Egr1, were significantly downregulated in the AD mice compared to the wild-type mice, while these transcription factors and target genes were upregulated after the treadmill exercise. These results confirm that treadmill activity attenuates atrophy and fibrosis in the skeletal muscle of AD mice, which is thus brought more similar to that of wild-type mice by regulating gene expression in the AD mice skeletal muscle, resulting in an improvement on the exercise ability of AD mice.

Exercise is a planned, noninvasive, low-cost, personalized physical activity and, most importantly, a physiologically natural way to be healthy [21]. Burgeoning clinical evidence suggests that exercise, as a nonpharmacological approach, has a positive effect on the prevention and treatment of cognitive decline in the aged population [22,23]. APP/PS1 transgenic AD mice suffer from learning–memory dysfunction and anxiety-like behavior, and our study findings indicate that a 12-week treadmill exercise regimen improved the originally low learning–memory ability of AD mice and led to anxiety relief, which is consistent with the results of current studies [24,25]. This further demonstrates the effectiveness of physical activity in alleviating AD. Additionally, given the unique advantages of physical activity, the mechanisms associated with improvement in the AD symptoms should be studied in greater depth in the future to increase its implementation in clinical practice.

Considering its weight, the skeletal muscle is the largest organ in the human body and is involved in maintaining body posture and performing autokinetic movements [26]. Kerr et al. immobilized the hindlimbs of female rats to inhibit their muscle function and found increased production of mitochondrial reactive oxygen species and elevated markers associated with amyloid production and cleavage in the hippocampus of the rats [27]. Numerous clinical studies and meta-analyses have also shown that sarcopenia increases the risk of cognitive impairment [28,29]. This suggests that unhealthy skeletal muscle may significantly contribute to or exacerbate cognitive impairment and that improving skeletal muscle function may be effective in improving the cognitive function of the brain. Thus, we explored the effects of exercise on skeletal muscle in the AD mice. In this study, we found that AD mice had reduced exercise ability, and further observation of the skeletal muscle structure revealed skeletal muscle atrophy, skeletal muscle fibrosis, and disorganized muscle fiber arrangement, suggesting that AD mice, similar to their AD human counterparts, have reduced exercise capacity due to the decline in skeletal muscle functions. The exercise ability of mice in the ADE group was improved, the atrophy and fibrosis of the skeletal muscle were reduced, and the muscle fiber arrangement became regular, suggesting that physical activity improved the cognitive function of the brain of AD mice. The treadmill exercise improved the skeletal muscle functions and exercise ability of AD mice, highlighting its effectiveness in preventing the decline in exercise ability and skeletal muscle functions of patients with AD.

Muscle atrophy occurs when protein degradation exceeds protein synthesis in the skeletal muscle [30]. The IGF-1 signaling pathway plays an important role in maintaining protein synthesis in muscle. After IGF-1 combines with the ligand, phosphorylated IGF-1R activates the intracellular junctional protein insulin receptor substrate-1 (IRS-1) and further induces the activation of the downstream PI3K/Akt pathway, which subsequently increases protein synthesis through the activation of mTOR [31]. In our study, we observed the inhibition of the IGF-1/PI3K/Akt/mTOR signaling pathway in skeletal muscle and a reduction in PI3K/Akt phosphorylation in the AD mice, whereas the treadmill exercise enhanced the IGF-1/PI3K/Akt/mTOR signaling pathway and promoted PI3K/Akt phosphorylation in both wild-type and AD mice, indicating the role of treadmill activity in improving protein synthesis in mouse skeletal muscle. We then investigated the UPS and ALP protein degradation systems. The results showed that the UPS-related factors Murf, MAFbx, and Fbxo32 were downregulated in the skeletal muscle of AD mice. After the intervention, their expression was elevated in the AD mice but decreased in wild-type mice, suggesting that exercise inhibited UPS in wild-type mice but promoted it in the AD mice. The ALP-related factors At9a, Lc3a, p62, and Beclin were downregulated in the skeletal muscle of AD mice and upregulated in both AD and wild-type mice after exercise, suggesting that exercise promoted ALP in both wild-type and AD mice. Skeletal muscle protein synthesis was reduced in the AD mice, as was protein degradation, while exercise promoted both protein synthesis and degradation. Exercise may have contributed to the modulation of a new balance of skeletal muscle protein synthesis and degradation in the AD mice, leading to enhanced health. Remarkably, we found similar results in our previous study of hepatic oxidative stress in the AD mice [8], which may be related to the originally lower metabolism of patients with AD and should be further investigated in the future.

RNA-Seq was used to explore the characteristics of gene changes during the intervention to improve the exercise ability of AD mice, and significant differences were observed in skeletal muscle gene expression between AD and wild-type mice (Figure 5C). The skeletal muscle gene expression in the AD mice was significantly changed after the exercise intervention (Figure 5D). The GO enrichment results show that compared with wild-type mice, the neuromuscular biological processes and cellular components in the skeletal muscle of AD mice had lower gene expressions, while after the exercise intervention, the gene expressions of the extracellular matrix, synaptic complexes, sarcolemma and other cellular components of skeletal muscle were upregulated in the AD mice; thus, these genes may play an important role in the amelioration of skeletal muscle atrophy in AD mice through physical activity. KEGG enrichment revealed that signaling pathways associated with skeletal muscle neuromuscular synapses (genes Gng3,Gng4, Grin1, and Slc17a7); hormone synthesis (genes Fos, Junb and Socs1); and inflammation (genes Fos, Fosb, Il1b, and Il27ra) were downregulated in the AD compared to the wild-type mice, and more pathways associated with diseases such as staphylococcus aureus infection (genes Defa23, Krt14, Krt25, and Krt33a), dilated cardiomyopathy and neurodegeneration of multiple diseases (genes App, Atp2a2, Fzd10, Nefm, Prnp, and Wnt2b) and AGE-RAGE signaling in diabetic complications (genes Egr1, Il1b, and Pim1) were upregulated. By contrast, after the exercise intervention, these disease-related pathways were downregulated in the skeletal muscle of AD mice, while signaling pathways associated with neuromuscular synapses, hormone synthesis, and inflammation were upregulated. Interestingly, six KEGG-enriched signaling pathways were downregulated and five were upregulated in the AD mice compared to the wild-type mice, and the expression levels of these pathways were reversed after the treadmill exercise. This suggests that the treadmill exercise intervention led to a more similar expression level in the skeletal muscle of AD and wild-type mice and that these genes may be potentially significant targets for improving the skeletal muscle functions of patients with AD through exercise. Physical activity led to the significant upregulation of ubiquitin-mediated proteolysis (genes Socs1 and Socs3), as well as signaling pathways involved in protein digestion and absorption (genes Col11a1 and Kcnj13) in the skeletal muscle of AD mice, suggesting the promotion of proteolysis, consistent with the qRT-PCR and protein immunoblotting results. In addition, inflammation-related pathways were downregulated in the skeletal muscle of AD mice, while exercise upregulated the pathways associated with inflammation regulation such as the IL-17 signaling pathway, TNF signaling pathway, and the inflammatory mediator regulation of TRP channels. Exercise has been widely known to induce the release of several inflammatory factors and promote a balance between anti- and pro-inflammatory in skeletal muscle, which contributes to metabolic and inflammatory regulation [32]. The skeletal muscles of AD mice may be in a metabolic and immunocompromised state, whereas a healthier balance between immunity and metabolism was achieved after the treadmill exercise. In addition, in the AD mice, exercise upregulated the IL27 genes, known for their role in regulating the immune system, promoting adipocyte thermogenesis and energy expenditure, reducing obesity, and ameliorating type 2 diabetes [33]. Interestingly, AD is known as type 3 diabetes because of its metabolic similarity to diabetes [34]. Therefore, IL27 may also be one of the important targets for improving AD metabolism through exercise.

TFs play a key role in regulating gene expression, thereby affecting various aspects of cellular physiology [20]. Because of the central role of TF activity, the dysregulation of TF activity is known to contribute to the development of many diseases, and several altered TFs have been detected in the brains of patients with AD [35]. Therefore, we further analyzed TFs. Interestingly, two transcription factors, Fosb and Egr1, as well as the corresponding target genes, Srrm4, Socs1, Il1b, Fosb, and Egr1, were significantly downregulated in the AD mice compared to the WT mice, whereas they were upregulated after the treadmill exercise. Reduced hippocampal Fosb in the AD mice increased cognitive deficits [36], exhibiting a consistent pattern with the results observed in skeletal muscle. In contrast, the inhibition of Egr1 expression in the AD hippocampus reduces Aβ pathology, improves cognitive performance, and reduces tau phosphorylation; thus, Egr1 has been recognized as a potential therapeutic agent for AD [37]. However, in the AD cases, exercise was found to be beneficial for enhanced Egr1 expression in skeletal muscle, an interesting finding that should be further investigated in the future to examine the different roles played by the same gene in various organs suffering AD.

Interestingly, Nagase et al. [38] immobilized AD mice with casts on both hindlimbs for 14 days to inhibit muscle function, which led to muscle atrophy. Muscle atrophy was found to accelerate the onset of cognitive deficits in AD by producing hemoglobin. This suggests that AD skeletal muscle health is closely related to the brain and that exercise improves AD cognitive function, but it is the skeletal muscle that is directly involved in exercise. The potential role of skeletal muscle in improving AD cognition through exercise is an open question that deserves further research. Our findings confirm that treadmill activity improves the exercise ability, as well as skeletal muscle functions, of AD mice and reveal potential changes in gene expression, which will provide a strong foundation for research in this direction and point to a possibly better efficacy of treatment strategies targeting these genes for AD prevention and rehabilitation.

## 4. Materials and Methods

### 4.1. Experimental Animals and Groups

Numerous studies have shown that rapid declines in estrogen and progesterone lead to a higher incidence of AD in women than in men [39,40]. In order to exclude the additional interference of estrogen, only male mice were used in this study. A total of 28 SPF-level 3-month-old male APP/PS1 double-transgenic mice and 28 SPF-level 3-month-old male C57BL/6J mice were purchased from Changzhou Cavens Laboratory Animal Co., Ltd. (Changzhou, China, License No. SCXK (Su) 2016-0010). APP/PS1 mice were randomly divided into a quiet group (ADC) and exercise group (ADE); likewise, C57BL/6J mice were randomly divided into a quiet group (WTC) and exercise group (WTE),with 14 mice in each group. They were kept in standard animal rooms of Hunan Normal University in strict accordance with animal welfare and ethics regulations.

### 4.2. Exercise Intervention

Following our previous research [41,42], the WTE and ADE groups were subjected to a treadmill exercise while increasing the speed from 7 m/min to 14 m/min in weeks 1 to 8 and then at a constant speed of 15 m/min in weeks 9 to 12. They exercised 5 days a week for 45 min each time.

### 4.3. Morris Water Maze

After 12 weeks of exercise, tests were conducted according to our previous methods [41,42,43], including 1 day of the swimming adaptation before the test, 5 days of the learning test, and 1 day of the memory test after removing the platform. Learning and memory abilities were assessed by calculating the change in the 5-day latent period, the number of times the specific position was crossed after the platform was removed, and the total distance of swimming in the water maze.

### 4.4. Open Field Test

Ten mice were randomly selected in each group. These mice were placed in an enclosed open field with a length, width, and height of 50 cm, the top of which was monitored using an infrared camera to record the free activities of each mouse for 5 min. The total moving distance of the mice in the open field, as well as the time and distance of their activities in the central area, were recorded to evaluate the anxiety level of these mice. During the testing process, a double-blind method was used to conduct the test, i.e., neither the operator of the experimental software nor the person who captured the animals for the experiment knew any information about the grouping of the animals.

### 4.5. Exercise Ability Test

**Figure 9:**
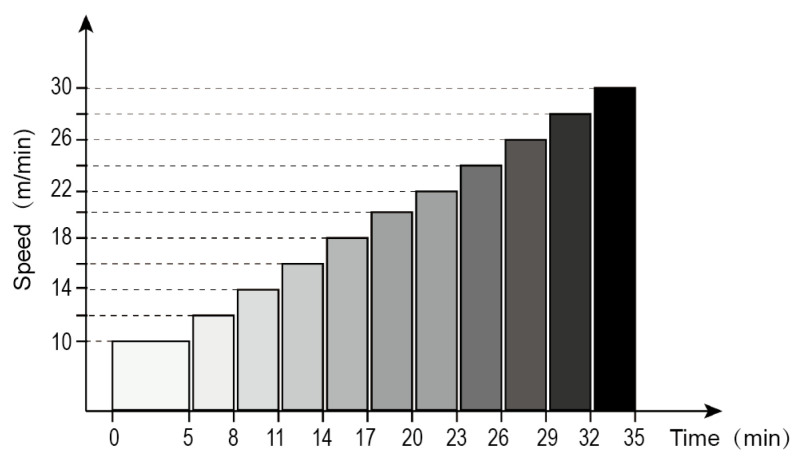
Program of exercise ability test.

Referring to research by Burch et al. [44], with certain modifications, a treadmill with increasing loads was used to assess the exercise ability. Four mice in each group were randomly selected to run on the zero slope of the platform at a speed of 10 m/min for 5 min. Then, the running speed was increased to 12 m/min, followed by an increase of 2 m/min every 3 min until exhaustion (Figure 9). Exhaustion was defined as the inability of the mice to continue running even after mechanical and electrical stimulation beyond 10 s. Then, the speed at the point of exhaustion, the total distance, and the total duration of exercise were recorded.

### 4.6. Tissue Collection

After behavioral experiments, the mice were fasted for 12 h, allowed free access to water, and then anesthetized with isoflurane the next morning. Quadriceps femoris muscle tissue samples were taken using cryopreservation tubes with liquid nitrogen rapid freezing and then stored at −80 °C for real-time PCR, Western blotting, and RNA-Seq experiments. In each group, 3 mice were randomly selected, and their quadriceps femoris muscle tissues were fixed in a 4% paraformaldehyde solution for 24 h~48 h. Then, sections were generated for HE staining, WGA staining, and Masson staining. Tissue collection was not performed on the mice participating in the exercise ability test.

### 4.7. Experimental Reagents, Instruments, and Methods

#### 4.7.1. HE Staining and Masson Staining

After conventional dehydration, vitrification, waxing, and embedding, 5 um slices were prepared, dewaxed, and hydrated, followed by hematoxylin–eosin staining and Masson staining, and, finally, the samples were dehydrated in graded ethanol, vitrified, and sealed. Quantitative statistics of HE-stained cross-sectional areas were performed using ImageJ Win32 (NIH, Bethesda, MD, USA).

#### 4.7.2. Wheat Germ Agglutinin (WGA) Staining

Antigen retrieval was performed on the paraffin sections of skeletal muscle. The samples were co-incubated with wheat germ agglutinin in dark conditions, followed by the DAPI staining of the cell nucleus, and the samples were imaged under a Leica DM2500 fluorescence microscope to observe the changes in the skeletal muscle tissues.

#### 4.7.3. Transmission Electron Microscopy

Small pieces of mouse skeletal muscle tissue were taken and fixed with an electron microscope fixative for 24 h. The tissues were dehydrated in graded ethanol and soaked in 100% acetone for 45 min at room temperature. Then, they were embedded in epoxy resin, cut transversely into 80 nm sections, and double-stained with 3% uranyl acetate–lead citrate. Finally, the samples were imaged under a transmission electron microscope [45].

#### 4.7.4. Real-Time PCR

Total RNA was extracted from mouse skeletal muscle tissues according to the Trizol reagent instructions (Waltham, MA, USA, ThermosFisher, 15596018). Miniamp PCR was used to perform reverse transcription according to the instructions of the TransGen Biotech Reverse Transcription Kit (Wuhan, China, Servicebio, G3337). The amplification system was configured in 20 μL according to the instructions of the Servicebio qPCR Kit (Wuhan, China, Servicebio, G3320), and amplification was accomplished using a Real-Time PCR instrument (Shanghai, China, BIO-RAD, 1855196). Finally, based on the detected Ct, the relative expression of the target gene mRNA was calculated using the 2^−∆∆CT^ method with GAPDH as the internal reference gene. Primer sequences were designed using Primer Bank (https://pga.mgh.harvard.edu/primerbank/ (accessed on 2 November 2023). The primer sequences for the genes are listed in Table 1.

#### 4.7.5. Western Blotting

Mouse quadriceps femoris muscle tissues from each group were taken and added to a mixture comprising RIPA lysis buffer (Wuhan, China, Servicebio, G2020) and the cocktail protease inhibitor (Wuhan, China, Servicebio, G2006) for grinding and centrifugation to obtain the supernatant, followed by protein quantification using a BCA Protein Concentration Assay Kit (Shanghai, China, Beyotime, P00125) according to the instructions. The samples were sequentially loaded, electrophoresed, transferred to the membrane, blocked, and incubated with the following primary antibodies—P-AKT (Changsha, China, AiFang, AF0908, 1:1000), AKT (Wuhan, China, Servicebio, GB111114, 1:1000, P-PI3K (CST, 4228, 1:1000), PI3K (Boston, MA, USA, CST, 4249, 1:50,000), MuRF (Wuhan, China, Proteintech, 21074-1-AP, 1:1000), Fbxo32 (Wuhan, China, Proteintech, 67172-1-Ig, 1:1000), and GAPDH (Wuhan, China, Servicebio, GB11002, 1:1000)—which were incubated overnight at 4 °C. The next day, they were washed for 10 min three times with PBST, incubated with a secondary antibody, and placed on a shaker for 1 h and 20 min at room temperature. After washing, they were imaged and photographed using an ECL Luminescence Kit (Wuhan, China, Servicebio, G2014) with the Tanon-5200 Gel System. The integral grayscale values were analyzed with ImageJ, and the relative protein expression statistics were determined.

#### 4.7.6. RNA-Seq

Three mice were selected from each group, and the total RNA was extracted from the quadriceps femoris muscle for paired sequencing using Illumina NovaSeq 6000 (Illumina, San Diego, CA, USA), developed by Majorbio Technology Co., Ltd. (Shanghai, China), and transformed into original sequenced reads. Transcriptome-sequenced reads were generated, and their quality was assessed using FastQC. After filtration and quality control on original data, a comparison of reference genomes, as well as homogeneity, saturation, and correlation analyses, were performed to determine the gene expression quantity and the differentially expressed genes (DEGs). The screening criteria for the DEGs were |log2FC| ≥ 1 and *p*-value ≤ 0.05. Gene Ontology (GO) and KEGG enrichment analyses of the DEGs were performed using the DAVID Database (accessed online: https://david.ncifcrf.gov/ (accessed on 5 May 2024)) and the package “ClusterProfiler” in R.

### 4.8. Statistical Analysis

All data are expressed as the means ± standard deviation (mean ± SD). Statistical analysis and plotting were performed using GraphPad Prism 8.0, with two-way ANOVA and post hoc multiple comparisons using the least significant difference (LSD). *p* < 0.05 is considered statistically significant, and *p* < 0.01 indicates a highly significant difference.

## 5. Conclusions

AD mice exhibit skeletal muscle atrophy and decreased exercise ability, and long-term treadmill activity enhances their exercise ability, reduces skeletal muscle atrophy, and improves protein synthesis and protein degradation, which may contribute to achieving a new balance between protein synthesis and degradation. Treadmill exercise leads to a similar expression level in the skeletal muscle of AD and wild-type mice by regulating the gene expression. The changes we observed in genes and pathways provide a research basis for the role skeletal muscle plays in treatment and cognitive improvement in patients with AD through physical exercise.

## Figures and Tables

**Table 1:** Primer sequences for target genes.

Gene	Forward Primer	Reverse Primer
IGF-1	ACCTGCCTGGGTGTCCAAAT	CGATAGGGACGGGGACTTCT
PI3K	CGAAACAAAGCCGAGAACC	GCAATGTTTGACTTCGCCAT
Akt	CTTCTATGGTGCGGAGATTGT	ACAGCCCGAAGTCCGTTATCT
mTOR	TTCCTGAACAGCGAGCACAA	TGCCAAGACACAGTAGCGGA
Fbxo25	AAGGTGTGACCCCTGTAGC	CCTCTTTTTGGCTGCGTATTCA
Fbxo32	CAGCTTCGTGAGCGACCTC	GGCAGTCGAGAAGTCCAGTC
MAFbx	CCACTTCTCAGAGCGGCAGA	CTTCTTGGGTAACATCGCACA
Murf	CCACTTCTCAGAGCGGCAGA	CTTCTTGGGTAACATCGCACA
lc3a	GACCGCTGTAAGGAGGTGC	CTTGACCAACTCGCTCATGTTA
At9a	GTTCGCCCCCTTTAATAGTGC	TGAACTCCAACGTCAAGCGG
P62	ATGTGAACATGGAGGGAAGA	GGAGTTCACCTGTAGATGGGT
Beclin	GAACTCTGGAGGTCTCGCT	CACCCAGGCTCGTTCTACC
GAPDH	CATGGCCTTCCGTGTTCCTA	CCTGCTTCACCACCTTCTTGAT

## Data Availability

The data used to support the findings of this study are available from the corresponding author upon request.
